# Novel thermoplastic splint for treating congenital auricular deformities with late initiation of treatment

**DOI:** 10.1016/j.jpra.2025.10.001

**Published:** 2025-10-17

**Authors:** Takuya Suzuki, Shinji Kumegawa, Maiko Inada, Yasuhiro Sakata, Yoshitaka Wada, Gen Yamada, Shinichi Asamura

**Affiliations:** Department of Plastic and Reconstructive Surgery, Wakayama Medical University, Wakayama, Japan

**Keywords:** Congenital ear deformity, Non-surgical treatment, Splint, Late initiation

## Abstract

Non-surgical treatment for congenital auricular deformities is commonly performed and has relatively favorable outcomes. However, delayed treatment could result in a poor response rate, eventually requiring surgical treatment. Effective non-surgical treatment for cases with late initiation of treatment is therefore required. We conducted a retrospective study of the treatment of congenital auricular deformity using a splint produced from a combination of thermoplastic resin and safety pins. Patients who visited our institution between March 2017 and March 2023 were included in the study. Treatment efficacy was determined from clinical photographs and statistically examined in relation to the initiation of treatments. We assessed how the timing of initiation of treatment affects the treatment response rate. Overall, 24 children (32 ears) were enrolled in our study, 22 of whom started treatment after 6 weeks of birth. Irrespective of the timing of initiation of such treatment, many patients responded to the treatments (29/32 ears). Treatment response rates were compared between two groups, depending on whether it was initiated before or after 12 weeks of age. However, there was no significant difference in the outcomes or duration of treatment according to the timing of its initiation. Our splint brought favorable results in our cohort, even when treatment was started after 12 weeks. The splint is inexpensive, possesses few adverse events and can be fine-tuned. Expanding the age range for non-surgical treatment is suggested to reduce the patient burden.

## Introduction

Congenital auricular deformities (CADs) are relatively common anomalies. Approximately half of all newborns reportedly possesses an auricular deformity.^1^^,^^2^ It will self-correct in some children, though residual deformity could result in cosmetic dissatisfaction and functional disability. There are two type of treatments: non-surgical treatment in the infants period, and surgery in childhood. Various non-surgical treatments for CAD have been reported, and they included a number of different materials for ear molding: tape,^3^ dental compound,^4^ wax,^5^ wire within a tube,^6^, ^7^, ^8^, ^9^ correction molding system,^10^, ^11^, ^12^, ^13^ urethane foam,^14^ and resin.^15^^,^^16^ High response rates have been observed in cases with early treatment initiation.^17^

Neonatal treatment responsiveness is due to maternal estrogen-dependent plasticity of the auricular cartilage.^18^ Delayed initiation of treatment is therefore thought to result in prolonged duration of treatment and decreased response rate.^10^^,^^19^^,^^20^ Expanding the limits of the initiation of non-surgical treatment for CAD could avoid surgical treatments with highly invasive and complication risks.^21^^,^^22^The currently required issue in the nonsurgical treatment of CAD is to establish effective corrective methods for cases in which treatment cannot be initiated earlier. Our therapeutic approach utilizes a novel splint capable of exerting a sufficient corrective force to overcome the increased resistance of less plastic auricular cartilage, with the aim of improving outcomes in cases of delayed treatment initiation. In this study, we conducted a case study of CAD treatment in our institution using novel thermoplastic splint and examined the effectiveness of the treatment, in particular, the relationship between the timing of treatment initiation and its effectiveness.

## Material and methods

### Patients

Patients with CAD who visited our institution for one of three types of deformity between March 2017 and March 2023 were included in this study. Excluded from the study were cases in which parents did not request treatment, cases that were lost to follow-up before the end of treatment, and cases lacking pre- and/or post-treatment photographs. The current study has been approved by the internal review board of Wakayama Medical University (approval number: 3969) and adheres to the STROBE guidelines. Written informed consent was obtained from the parents for publication of clinical photographs in the article.

### Types of auricular deformities

**Figure 1:**
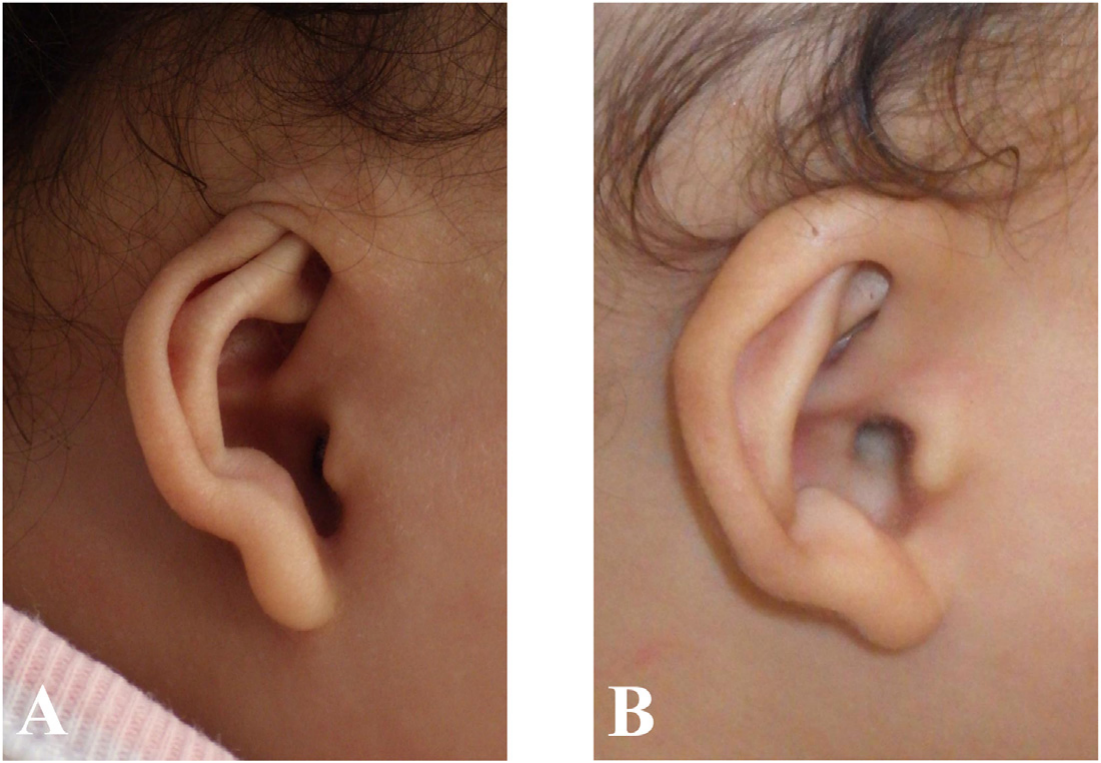
Images of the cryptotia before (A) and after (B) treatment. The treatment was initiated at 125 days of age; duration of treatment was 147 days.

**Figure 2:**
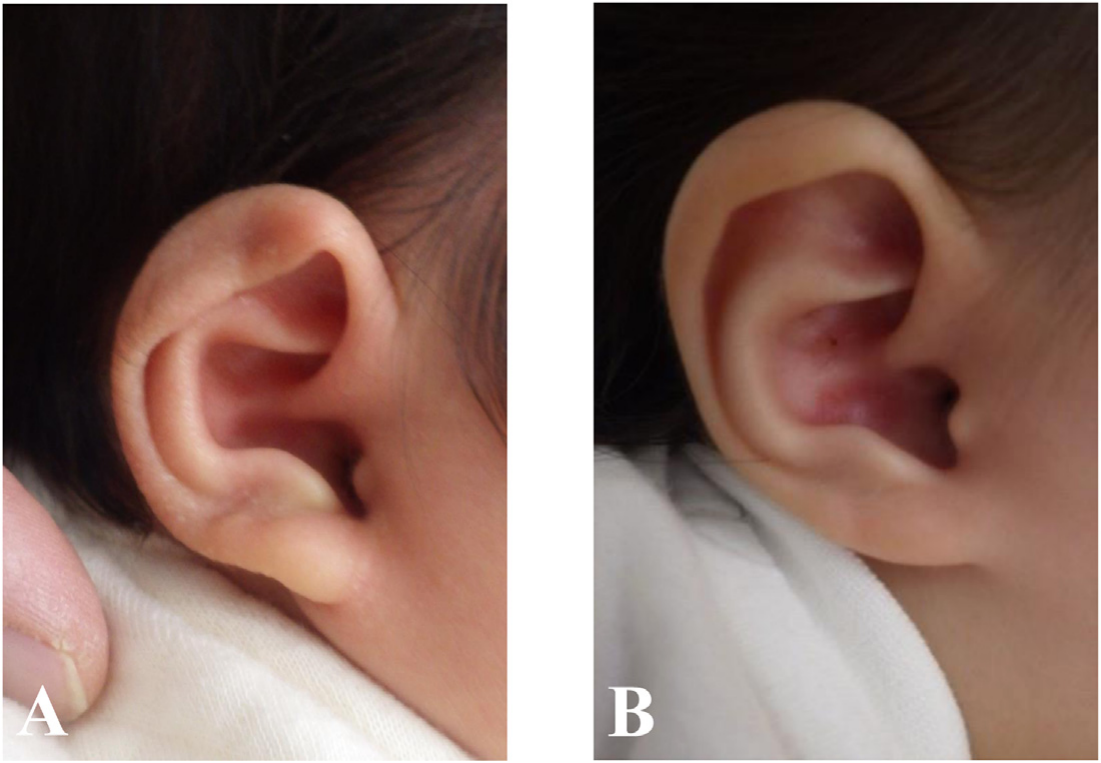
Images of the lidding ear deformity before (A) and after (B) treatment. The treatment was initiated at 69 days of age; duration of treatment was 209 days.

**Figure 3:**
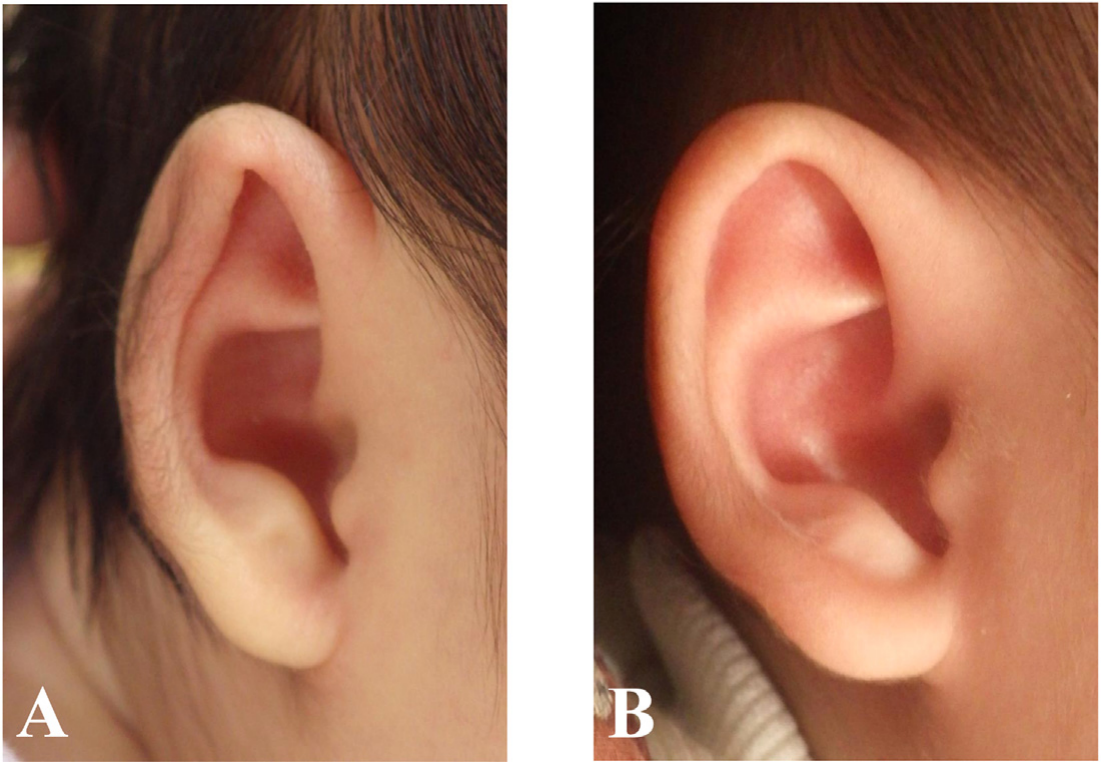
Image of the helical rim deformity before (A) and after (B) treatment. The treatment was initiated at 66 days of age; duration of treatment was 126 days.

We performed nonsurgical treatment of three types of auricular deformities. Cryptotia is a deformity in which the upper pole of the auricle is buried by the skin of the temporal region (Figure 1A). A lidding ear deformity is distortion of the cartilage and skin that comprises the helical rim and scapha (Figure 2A). A helical rim deformity is characterized by the folding over of the upper pole of auricle (Figure 3A).

### Novel splint

**Figure 4:**
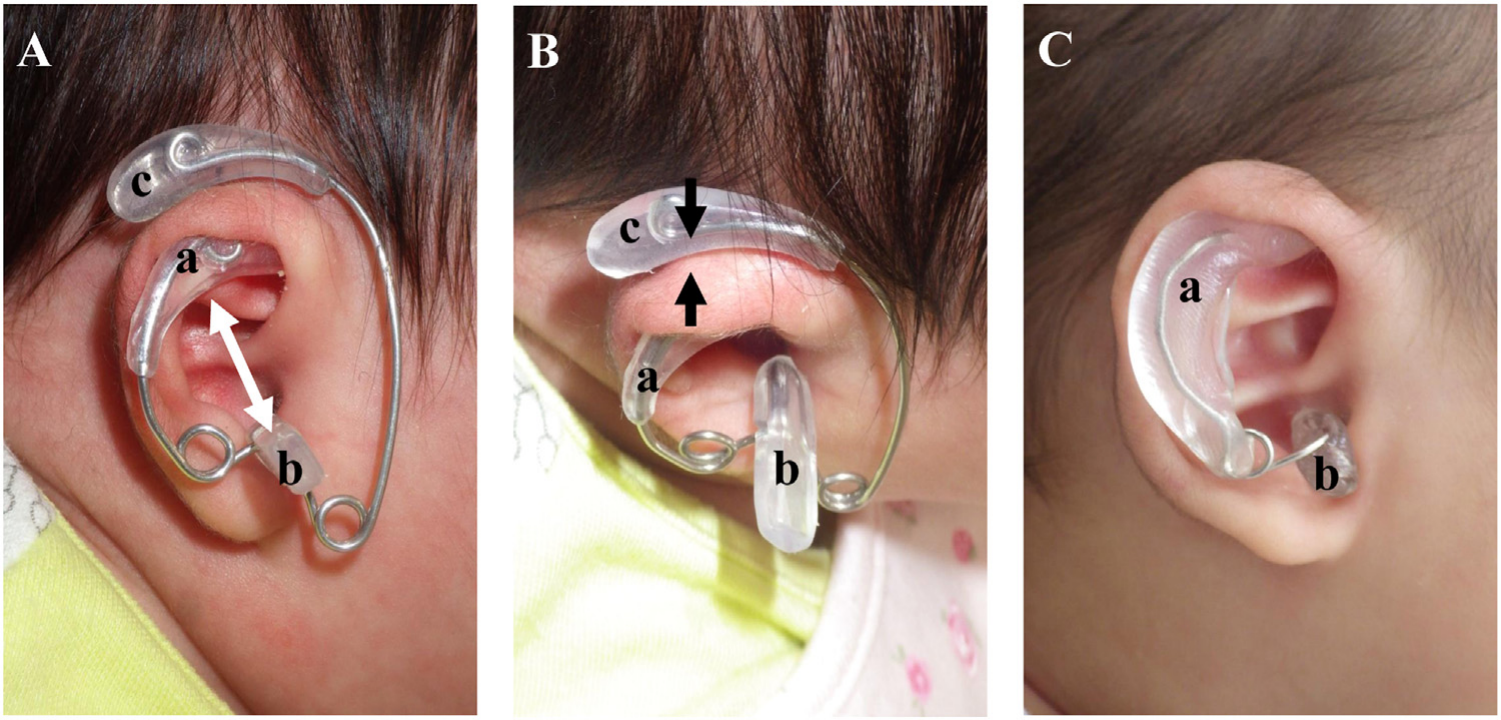
Image of the splint applied to the ear (A and B). Image of a splint consisting of two fillers applied to the ear (C). Between the scaphoid fossa and incisura intertragic is extended by filler (white arrow). The upper part of auricle is pinched by the splint (black arrows). a: scaphoid filler, b: incisura intertragica filler, c: auriculocephalic sulcus filler.

The novel splint used in this study consists of a framework fabricated by bending safety pins and thermoplastic resin (Erkoflex:ERKODENT, Pfalzgrafenweiler, Germany) that fills the scaphoid, incisura intertragica, and auriculocephalic sulcus (Figure 4). Safety pins are bent to match the shape of the auricle, and unnecessary portions are removed to fit the size of each auricle. Thermoplastic resin is modulated by a heat gun and bonded to the framework and subsequently molded to fit each part of the auricle (Supplemental video 1). To maintain the corrected auricular contour, the splint was adjusted by thermoforming the plastic resin and bending its supporting framework. To prevent pressure-induced skin injuries, the skin underlying the splint was inspected for any discoloration, such as erythema or blanching, at the time of each splint adjustment. This entire procedure was typically completed within 20 min in the examination room. The splint was produced without the filler for auriculocephalic sulcus depending on the degree of deformity (Figure 4C).

### Treatment procedure

Patients were examined by a physician and prosthetist, and the treatment with a splint was initiated as early as the same day. The actual fabrication and adjustments were performed by the prosthetist, and the final fitting was approved by a physician. The same procedure was applied to all follow-up adjustments. The splint was applied for several hours per day and also for longer periods of time after confirming there are no complications. The splint was applied and removed by their parents. In general, the splint is applied without tape, although it is fixed with tape for support in instable cases. After the start of treatment, patients were re-examined one or two weeks later to check for skin irritation and other complications. The child was examined regularly, and the splint was adjusted according to the growth of the auricle. The treatment was terminated when the auricular deformity was thought to be improved and maintained its shape, when the plasticity of the auricle decreased and the treatment was no longer though to be effective, or for the cases not allowing to continue to wear the splint because the patients remove it.

### Assessment and data collection

The type of deformities and the effect of treatment were evaluated by referring to photographs before and after the treatment. The outcomes were rated as previously reported: excellent (almost normal ear shape), good (near normal shape with some degree of abnormality) and poor (slight or no improvement).^23^ Evaluations were performed blindly by three plastic surgeons. Scores “good” and “excellent” were defined as a response to treatment. Sex, age at the start of treatment, and duration of treatment were collected from medical records.

### Statistical analysis

An analysis of variance was performed to compare the timing of treatment initiation and duration of treatment between each type of deformity. Fisher’s exact tests were performed to compare treatment outcomes between each deformity and to examine the relationship of treatment outcome by age at initiate treatment. Pearson's correlation coefficient was calculated to investigate the relationship between the age initiating the treatment and the duration of treatment. Statistical analysis was conducted with JMP pro 17.2 (SAS Institute Inc. Cary, NC, USA).

## Results

### Patient demographics

Overall, 30 children (39 ears) were treated with the splint, though six children were excluded owing to match the pre-determined exclusion criteria. Finally, 24 children (32 ears) were enrolled in the study. Patient demographics are summarized in Table 1. The mean age of treatment initiation was 92.9 days, and the mean duration of treatment was 136.4 days. Complications associated with treatment included skin irritation in one case, which improved with ointment treatment, and treatment could be continued.

**Table 1:** Patient demographics (32 ears from 24 patients).

Category	Value
Gender (*N* = 24)	
Male	8
Female	16
Ear (*N* = 32)	
Right	17
Left	15
Type of deformity (*N* = 32)	
Cryptotia	11
Helical rim deformity	12
Lidding ear deformity	9
Age at initiate treatment (*N* = 32)	
<6 weeks	3
6–12 weeks	16
>12 weeks	13
Mean (days)	92.9 ± 50.9
Range (days)	25 - 254
Treatment duration (*N* = 32)	
Mean (days)	136.4 ± 62.8
Range (days)	21 - 244

Means are presented in the form of value ± standard deviation. SD, standard deviation.

### Types of the ear deformity

**Figure 5:**
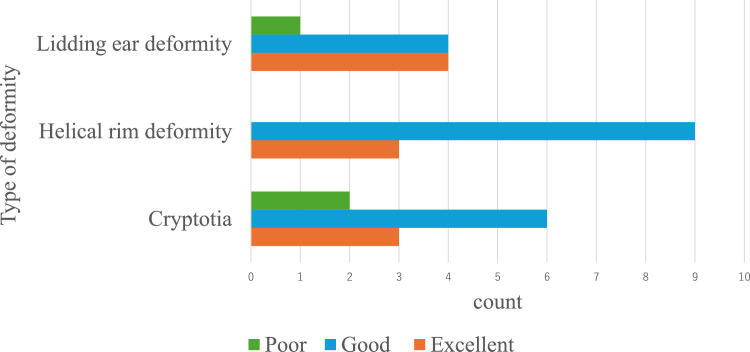
Outcome by the types of deformities; No significant response rate among the types of deformities are observed (*p* = 0.81 Fisher’s exact test).

Age at the start of treatment and duration of treatment did not differ significantly according to the type of deformity (Table 2). Representative cases of cryptotia, lidding ear deformity and helical rim deformity are shown in Figure 1, Figure 2, Figure 3, respectively. Evaluations of the treatment are shown in Figure 5. The treatment response rate was favorable, with many cases (29/32 ears) responding to treatment (judged as “excellent” or “Good”). No significant differences in treatment effects among types of deformity were observed (*p* = 0.81 Fisher’s exact test).

**Table 2:** Timing of treatment initiation and duration of treatment by the types of deformities.

	All		Cryptotia		Helical rim deformity		Lidding ear	η^2^	*p*-value^a^
	*n* = 32		*n* = 11		*n* = 12		*n* = 9		
Initiation (day)								0.0016	0.98
Mean	92.9		93.7		94.6		89.8		
SD	50.9		67.5		50.2		29.4		
Duration (day)								0.13	0.13
Mean	136.4		121.7		123.0		172.1		
SD	62.8		65.0		60.5		54.3		

a ANOVA test.

### Initiation of the treatment

To examine the relationship between the timing of treatment initiation and treatment outcome, analysis was conducted by dividing the patients into two groups based on whether treatment began before or after 12 weeks of age. No significant difference in treatment outcome was observed between the two groups (Table 3). We also performed an analysis of the relationship between treatment initiation and duration of treatment and there was no significant correlation between them (correlation coefficient: 0.10, 95 % confidence interval: −0.26 to 0.43).

**Table 3:** Relationship between the timing of treatment initiation and treatment efficacy.

	>12 weeks	<12 weeks	total
Improve	11	18	29
No improve	2	1	3

total	13	19	32

Patients were divided into two groups at around 12 weeks of treatment initiation for analysis. No significant differences of outcomes were found between the two groups (*p* = 0.36 Fisher's exact test). Improve: judged as “excellent” or “good,” No improve: judged as “poor.”

## Discussion

### The material and the structure of the splint

Our splint consists of two materials: a framework composed of safety pins and a filler made of thermoplastic resin. The thermoplastic resin part as filler prevents strong force from being applied to a single point. We suggest this filler could be useful in the correction of lidding ear deformity and helical rim deformity by expanding the scaphoid. The frame can be adjusted to the changes in auricular morphology as a result of growth by bending the frame or by adjusting the size and shape of the resin.

Auricular molding equipment has been classified into four categories: auriculocephalic sulcus type, scapha-and-concha type, sandwich type, and fill and fix type.^24^ Our splint combines the scapha and concha type and the auriculocephalic type in one device. By applying it, the space between the scaphoid fossa and incisura intertragica is extended, the upper part of the auricle is pinched, and the auriculocephalic sulcus is pressed to correct the auricular deformity (Figure 4). This device can therefore be claimed to possess characteristics similar to the fill and fix type. Fillers to the auriculocephalic sulcus are utilized when particularly strong correction is needed. In many cases, correction with filler only in the scaphoid and incisura intertragica has been effective.

In cases where initiation of treatment is delayed, stronger correction should be applied because of the decline in plasticity of the auricular cartilage. Tape and urethane foam are accessible and easy to process, but they cannot apply a strong force to the auricle, and tape may be difficult to fix them firmly because of hair. Resins, waxes, and compounds may require tapes for stable fixation. The commercial molding system shows favorable results with early treatment.^10^ However, hair may need to be shaved for application in some cases.

The following characteristics have been reported for an ideal splint: light and inexpensive, easy and quick to fabricate, adjustable to complicated contours, easy to apply and remove, easy to reform, and adjustable to the degree of correction.^25^ The current splint fulfills all of these requirements except that it is not particularly quick and easy to fabricate. The EVA resin used in our splint is the same as that used in denture bases and mouthpieces. It is an inexpensive material, along with the safety pins used in the frames. The current splint is easy to apply and thus can be applied and removed by the parents.

The ease of application and removal by caregivers was a notable advantage. The ability for caregivers to easily reapply the device likely improved overall treatment compliance, especially following instances of self-removal by the child. Additionally, our protocol included the judicious use of adhesive tape to augment fixation when necessary, thereby aiming to maximize the corrective effect. Ease of application and removal is also useful for continuing treatment and for early detection of skin irritation.

Nevertheless, our splint has limitations. Deformities such as shallow scaphoid fossa or a conchal crus were found to be difficult to manage effectively. The treatment of these specific anomalies likely requires devices with alternative corrective mechanisms, underscoring the importance of matching the splint to the individual deformity.

### Age at the initiation of molding

The effectiveness of nonsurgical treatment for auricular deformities has been frequently reported,^17^ and early treatment is desirable in terms of its duration and efficacy. Circulating estrogen levels, which are involved in the plasticity of the auricular cartilage,^18^ return to the level of the children at 6 weeks of age, many authors thus recommend starting treatment within 6 weeks of birth.^10^^,^^19^^,^^20^ In the current study, almost all patients started treatment after 6 weeks of age (29/32 ears) with satisfactory treatment results. Comparison of the two groups, those who started treatment before and after 12 weeks showed no significant difference in treatment efficacy, indicating that treatment with our splint may be effective even for patients who cannot start treatment earlier.

Delay in initiating treatment has been associated with a longer duration of treatment.^11^, ^12^, ^13^ In the current study, however, no correlation was found between the timing of treatment initiation and the duration of treatment. Although the differences in treatment duration among deformity types did not reach statistical significance (*p* = 0.13), the large effect size (η²=0.13) indicates a clinically relevant trend that warrants discussion. Specifically, our data showed a tendency for lidding ear to require a more prolonged course of treatment compared to other types. This discrepancy between a non-significant p-value and a large effect size is likely a consequence of insufficient statistical power due to our modest sample size. Another potential factor is that many patients come to our clinic from relatively distant locations, and the interval between follow-up examinations is longer in these cases. We therefore recommend that they wear a splint for the period between follow-up examinations. This could be also because patients are instructed to continue wearing the splint after the deformity has been corrected to prevent recurrence in some cases. For the same reason, the overall duration of splinting could be also longer.

### Limitation of this study

In the current study, the determination of treatment efficacy was exclusively from the physician's point of view. Parental evaluation can also be an important aspect of CAD treatment, it would be therefore desirable to record evaluations from the part of parents. There are also no strict criteria for the termination of treatment, so it may be necessary to consider completion criteria for this splint in order to determine a more accurate treatment duration. Furthermore, treatment compliance was not formally assessed, as it depended on the caregivers' recall of splint wear, which was often unreliable.

## Conclusion

We treat CADs with splint composed of thermoplastic resin and a safety pin. The splint is inexpensive, easy to apply, and can be adjusted according to changes in auricular morphology. The current splint, which can combine expansion and retraction, is considered as effective for CADs with delayed initiation of treatment, which has been conventionally difficult.

## Ethical approval

The current study has been approved by the internal review board of Wakayama Medical University (approval number: 3969).

## Declaration of competing interest

The authors declare that there are no relevant conflicts of interest.

## Appendix Supplementary materials

Image, video 1
